# Mitochondrial transplantation combined with coenzyme Q_10_ induces cardioprotection and mitochondrial improvement in aged male rats with reperfusion injury

**DOI:** 10.1113/EP091358

**Published:** 2024-03-13

**Authors:** Soleyman Bafadam, Behnaz Mokhtari, Manoucheher Seyedi Vafaee, Zohreh Zavvari Oscuyi, Samira Nemati, Reza Badalzadeh

**Affiliations:** ^1^ Molecular Medicine Research Center, Biomedicine Institute Tabriz University of Medical Sciences Tabriz Iran; ^2^ Department of Physiology, Faculty of Medicine Tabriz University of Medical Sciences Tabriz Iran; ^3^ Drug Applied Research Center Tabriz University of Medical Sciences Tabriz Iran; ^4^ Psychiatry Research Unit Odense Denmark; ^5^ Department of Nuclear Medicine Odense University Hospital Odense Denmark; ^6^ Physiology Research Center Semnan University of Medical Sciences Semnan Iran

**Keywords:** ageing, coenzyme Q_10_, mitochondrial transplantation, myocardial ischaemia/reperfusion injury

## Abstract

Ischaemic heart diseases (IHD) are among the major causes of mortality in the elderly population. Although timely reperfusion is a common treatment for IHD, it causes additional damage to the ischaemic myocardium known as ischaemia–reperfusion (IR) injury. Considering the importance of preventing reperfusion injuries, we aimed to examine the combination effect of mitochondrial transplantation (MT) and coenzyme Q_10_ (CoQ_10_) in myocardial IR injury of aged male rats. Seventy‐two aged male Wistar rats were randomly divided into six groups: Sham, IR, CoQ_10_, MT, combination therapy (MT + CoQ_10_) and vehicle. Myocardial IR injury was established by occlusion of the left anterior descending coronary artery followed by reopening. Young male Wistar rats were used as mitochondria donors. Isolated mitochondria were injected intraventricularly (500 µL of a respiration buffer containing 6 × 10^6^ ± 5 × 10^5^ mitochondria/mL) in MT‐receiving groups at the onset of reperfusion. CoQ_10_ (10 mg/kg/day) was injected intraperitoneally for 2 weeks before IR induction. Twenty‐four hours after reperfusion, haemodynamic parameters, myocardial infarct size (IS), lactate dehydrogenase (LDH) release and cardiac mitochondrial function (mitochondrial reactive oxygen species (ROS) generation and membrane potential) were measured. The combination of MT and CoQ_10_ improved haemodynamic index changes and reduced IS and LDH release (*P *< 0.05). It also decreased mitochondrial ROS generation and increased membrane potential (*P *< 0.05). CoQ_10_ also showed a significant cardioprotective effect. Combination therapy displayed greater cardioprotective effects than single treatments. This study revealed that MT and CoQ_10_ combination treatment can be considered as a promising cardioprotective strategy to reduce myocardial IR injury in ageing, in part by restoring mitochondrial function.

## Abstract (highlights)

- **What is the central** **question of this study?**
  Can mitochondrial transplantation (MT) along with coenzyme Q_10_ (CoQ_10_) administration induce greater cardioprotection following ischaemia–reperfusion (IR) injury in aged male rats?
- **What is the main finding and its importance?**
  MT–CoQ_10_ combination therapy improved cardiac function and diminished infarct size in aged IR rats, which was associated with increased mitochondrial membrane potential and decreased reactive oxygen species production.

## 1 INTRODUCTION

Cardiovascular diseases have a high prevalence and mortality rate worldwide. Among them, ischaemic heart diseases (IHD) are considered the most common cause of mortality (Wong, 2014; Zhu et al., 2014). IHD occurs as a result of insufficient blood flow to the myocardial tissue, which causes myocardial cell death (Severino et al., 2020). Although reperfusion therapy is the main treatment for IHD, it increases mortality in patients with myocardial infarction by causing additional damage to the myocardial cells and increasing the infarct size, a process called ischaemia–reperfusion (IR) injury (Hausenloy & Yellon, 2013). Several pathophysiological mechanisms are involved in myocardial IR injury including oxidative stress, intracellular calcium accumulation, mitochondrial dysfunction, disruption of metabolic processes and depletion of ATP (Gunata & Parlakpinar, 2021; He et al., 2022; Saeid et al., 2018).

Ageing is known as a main risk factor for IHD (Niccoli & Partridge, 2012). Most patients with cardiac ischaemia are old and exposed to cardiovascular risk factors and comorbidities such as diabetes, hyperlipidaemia and high blood pressure (Feyzizadeh & Badalzadeh, 2017). Ageing decreases the normal physiological function of the heart as well as the capacity of cardiomyocytes to maintain homeostasis, and thus increases the susceptibility of the myocardium to ischaemic damage (Pomatto & Davies, 2017; Rodrigues et al., 2020). With increasing age, mitochondria undergo structural and functional changes including a decrease in the integrity, dynamics and respiratory chain activity, deformations (swelling and shrinkage), DNA mutation, and an increase in reactive oxygen species (ROS) production (Schneider et al., 2020; Wu et al., 2019). The age‐related mitochondrial dysfunction renders the cardiovascular system more susceptible to pathological stress. Cardiac mitochondrial damage and oxidative stress are central mechanisms in the development of cardiomyocyte death following IR injury, especially when associated with ageing (Kelm et al., 2020; Subramani et al., 2020). Accumulation of dysfunctional mitochondria and oxidative stress during ageing promotes the events caused by ischaemia and reperfusion, and also reduces the cardioprotective efficacy of therapeutic interventions (Wang et al., 2013).

Mitochondria‐directed therapeutic interventions are considered as a promising approach for cardioprotection against IR injury during ageing (Marin et al., 2021). Previous studies have shown that mitochondrial transplantation (MT), the replacement of damaged mitochondria with healthy viable mitochondria, improves cardiac contractile function, increases the survival of damaged cardiomyocytes and reduces the infarct size (Cowan et al., 2016; Guariento et al., 2020; Masuzawa et al., 2013). On the other hand, the reduction of mitochondrial function during ageing can also be attributed to the reduction or loss of various mitochondrial boosters and cofactors such as coenzyme Q_10_ (CoQ_10_). CoQ_10_ is a small lipophilic molecule present in all biological membranes, mainly found in the mitochondrial inner membrane, where it plays a key role in the mitochondrial electron transport chain (Awad et al., 2018). CoQ_10_ acts as a potential antioxidant, stabilizes plasma membrane, protects membrane lipids against peroxidation, and recycles other antioxidant molecules such as alpha‐tocopherol and ascorbate (Gutierrez‐Mariscal et al., 2021). It also improves mitochondrial respiration, biogenesis, and ATP production, and reduces apoptosis (Lu et al., 2017). Since the intracellular content of CoQ_10_ decreases during ageing as well as myocardial IR injury, administration of CoQ_10_ can exert cardioprotective effects in these conditions (Aaseth et al., 2021).

Considering the key role of CoQ_10_ in the normal function of mitochondria as well as the reduction of its content in aged cardiomyocytes, we hypothesized that its administration along with MT could be useful in boosting the function of transplanted as well as residual mitochondria following IR injury. Therefore, this study aimed to investigate the effect of MT in combination with CoQ_10_ pretreatment on cardioprotection and mitochondrial function in the heart of aged male rats subjected to IR injury.

## 2 METHODS

### 2.1 Ethical approval

All animal experiments were conducted in line with the NIH *Guide for the Care and Use of Laboratory Animals* (publication no. 85‐23, revised 1985) and confirmed by the Ethics Committee of the Tabriz University of Medical Sciences (Ethics approval code: IR.TBZMED.REC. 1399.425).

### 2.2 Animals

Seventy‐two aged male Wistar rats (22 ± 2 months old, weighing 450 ± 50 g) and 10 young Wistar rats (6 weeks old, weighing 200 ± 30 g) were purchased from the animal centre of Tabriz University of Medical Science (Tabriz, Iran). Animals were housed in standard cages under controlled temperature (22 ± 2°C) and 12 h–12 h dark–light cycles with free access to drinking water and standard food.

### 2.3 Experimental design

Aged rats were randomly divided into six groups (12 in each group): (1) Sham group (receiving chest surgery without IR), (2) IR group (receiving IR), (3) MT group (receiving IR plus mitochondrial transplantation), (4) CoQ_10_ group (receiving IR plus CoQ_10_), (5) MT + CoQ_10_ group (receiving IR plus combination therapy), and (6) Vehicle group (receiving IR plus 1% dimethyl sulfoxide (DMSO) and respiration buffer). CoQ_10_ (Sigma‐Aldrich, Germany) was injected intraperitoneally daily for 2 weeks at a dose of 10 mg/kg dissolved in 0.1 mL of 1% DMSO before the establishment of the IR model (Eleawa et al., 2015). In MT‐receiving groups, 500 µL respiration buffer comprising approximately 6 × 10^6^ mitochondria/mL was injected into the left ventricle (LV) chamber of the heart within 2 min at the onset of reperfusion (Jabbari et al., 2020). All IR receiving groups were subjected to 30 min of ischaemia and 24 h reperfusion. Throughout various stages of the surgical procedure and subsequent recovery, two rats in each group died due to surgical failures, unsuccessful anaesthesia recovery, improper catheterization, or post‐surgical complications. Following the assessment of both surgical and postoperative losses, 10 rats remained in each group. Among these, four rats were randomly assigned for determining the infarct size, while the remaining six rats were allocated for assessing haemodynamic and biochemical parameters. LV samples were freshly used for mitochondrial function assessment. Cardiac blood samples were collected, and after centrifugation, sera were separated and stored at −80°C until lactate dehydrogenase (LDH) measurement.

### 2.4 Establishment of IR

The myocardial IR injury model was induced in rats based on a previous report (Ahmed et al., 2011). First, the animals were anaesthetized using intraperitoneal injection of ketamine–xylazine (80/10 mg/kg). Next, they were intubated and ventilated using a mechanical ventilator. The animal was placed in the supine position and a heating pad was placed under it to maintain the animal's body temperature in a physiological state (temperature 37 ± 1°C). The rat's chest was opened under sterile conditions to expose the heart. To create regional ischaemia and myocardial infarction, the left anterior descending coronary artery (LAD) was closed 2–3 mm under the left atrial appendage using a 6‐0 suture and a legator for 30 min. After 30 min of ischaemia, the ligature was opened for blood reperfusion and the chest was closed and sutured. Immediately at the onset of reperfusion, mitochondria were injected into the LV chamber of animals in MT and MT + CoQ_10_ groups. After the completion of the surgical procedure, ketoprofen (10 mg/kg) was injected intraperitoneally to induce analgesia in sham and IR receiving animals.

### 2.5 Mitochondrial extraction

Ten healthy male rats were used for mitochondrial isolation. Animals were anaesthetized with ketamine and xylazine (80/10 mg/kg). The skin of the pectoral area was opened and a piece of pectoralis major muscle was removed and immediately harvested in mitochondrial isolation buffer at 4°C, comprising (mM): 70 sucrose, 200 mannitol, 2 EDTA and 10 HEPES, pH 7.4. The homogenate was transferred to a 2 mL tube and centrifuged at 1200 *g* for 10 min at 4°C. The supernatant was then collected and transferred to a 1.5 mL tube and centrifuged again at 12,000 *g* for 10 min at 4°C. Finally, the supernatant was discarded and the remaining pellet, containing the extracted mitochondria, was resuspended in 100 µL of storage buffer. The storage buffer composition was (mM): 250 sucrose, 1 ATP, HEPES, 5 sodium succinate, 0.08 ADP, 1 dithiothreitol, and 2 K_2_HPO_4_ (Mousavi et al., 2021). The number of extracted mitochondria was counted using the haemocytometry method. Subsequently, a mitochondrial concentration of 6 × 10^6^ ± 5 × 10^5^ mitochondria/mL was prepared, and 500 µL of the prepared compound was injected into the LV chamber of rats in MT and MT + CoQ_10_ groups. Finally, while the mitochondria donor rats were still under deep anaesthesia, they were killed by cervical dislocation.

### 2.6 Confirmation of mitochondrial homing in cardiac tissue

In order to ensure injected mitochondria are entered into cardiac tissue, in a separate pilot study, isolated mitochondria from healthy rats were incubated with 50 ng/mL MitoTracker Red (Thermo Fisher Scientific, Waltham, MA, USA) at 37°C and injected into the LV chamber of the aged rats. Twenty‐four hours later, the heart was removed and tissue sections were prepared and examined by fluorescence microscopy.

### 2.7 Haemodynamic measurement

Twenty‐four hours after reperfusion, six animals of each group were anaesthetized by ketamine and xylazine (80/10 mg/kg), and the carotid artery was catheterized for haemodynamic measurements. A PE50 pressure–volume catheter, connected to a pressure transducer and PowerLab system (ADInstruments, Bella Vista, NSW, Australia), was placed in the right carotid artery. First, arterial systolic and diastolic pressures were recorded for 20 min. Afterward, the catheter was gently guided into the LV to measure LV functional parameters, including heart rate, LV end‐diastolic pressure (LVEDP), LV developed pressure (LVDP) and LV contractility (±d*P*/d*t*). Data were analysed by LabChart 7.3 software (ADInstruments). Upon completion of the measurements, the hearts were removed under deep anaesthesia and animals were killed by exsanguination. The isolated hearts were subsequently utilized for the evaluation of mitochondrial function and the blood samples of these animals were also collected for LDH measurement.

### 2.8 Measurement of LDH release

A LDH Assay Kit (Pars Azmoon Co., Karaj, Iran) and an autoanalyser device (Alcyon 300, Abbott Labs, Santa Clara, CA, USA) were used to determine the level of LDH in blood serum samples collected from six rat in each group. The measurement was conducted using the colorimetric method according to the manufacturer's instructions, and the results are reported as IU/L.

### 2.9 Evaluation of myocardial infarct size and area at risk

Myocardial infarct size and area at risk (AAR) were assessed by the Evans blue/2,3,5‐triphenyltetrazolium chloride (TTC) staining method as previously described (Yu et al., 2021). Twenty‐four hours after reperfusion, four rats of each group were anaesthetized with ketamine/xylazine (80/10 mg/kg). The LAD was reoccluded and 2 mL of 2% Evans blue solution (Sigma‐Aldrich) dissolved in phosphate‐buffered saline (PBS) was immediately infused via the tail vein to determine AAR (unstained area in blue). Then, the hearts were removed and animals were killed by exsanguination under deep anaesthesia. After freezing, the heart was sectioned into 2 mm‐thick transverse slices. TTC staining was used to determine myocardial infarct size. The tissue slices were incubated in 1% TTC solution (Sigma‐Aldrich) dissolved in PBS (pH 7.4) at 37°C for 30 min. Then, slices were fixed in 10% formalin overnight. Stained slices were pictured by a digital camera. The AAR and infarct size were measured and analysed by ImageJ software (NIH, Bethesda, MD, USA). AAR and infarct size were expressed as a percentage of the total area and AAR area respectively.

### 2.10 Mitochondrial function assessment

To evaluate mitochondrial function in heart tissue, mitochondrial ROS generation and membrane potential were measured, as described previously (Bayrami et al., 2018). For this purpose, after completing the haemodynamic measurement, the hearts of four rats of each group were removed and mitochondria were extracted from the LV tissue according to the above‐mentioned technique, and the parameters were then measured.

#### 2.10.1 Mitochondrial ROS generation

Mitochondrial ROS production was detected using a DCFDA‐Cellular ROS Detection Assay Kit (ab113851, Abcam, Cambridge, UK). First, 40 µL dichlorodihydrofluorescein diacetate (DCFDA) dye was added to the extracted mitochondria and incubated at 37°C in the dark for 30 min. DCFDA is oxidized to 2′,7′‐dichlorofluorescein in the presence of ROS. Next, 2′,7′‐dichlorofluorescein fluorescence intensities were measured using a fluorescence microplate reader (Ex/Em = 485/535 nm), and the ROS level was determined as a percentage of the control after subtracting the background.

#### 2.10.2 Mitochondrial membrane potential

The JC‐10 Assay Kit (Sigma‐Aldrich, cat. no.: MAK159) was used to measure mitochondrial membrane potential following the manufacturer's instructions. First, JC‐10 dye was added to the extracted mitochondria (2 µg/mL) and incubated in the dark for 20 min at 37°C. The red fluorescence of JC‐10 (in its aggregate form) was excited at 525 nm and its emission was detected at 590 nm. The green fluorescence of JC‐10 (in its monomeric form) was excited at 485 nm, and its emission was detected at 530 nm. The ratio of red to green indicates the mitochondrial membrane potential.

### 2.11 Statistical analysis

All data were reported as the mean and standard deviation (SD) and analysed by one‐way ANOVA. To determine the statistical significance between groups, Tukey's post hoc test was applied. All analyses were performed using IBM SPSS Statistics 26 (IBM Corp., Armonk, NY, USA) and *P*‐values less than 0.05 were considered statistically significant.

## 3 RESULTS

### 3.1 Homing

**FIGURE 1:**
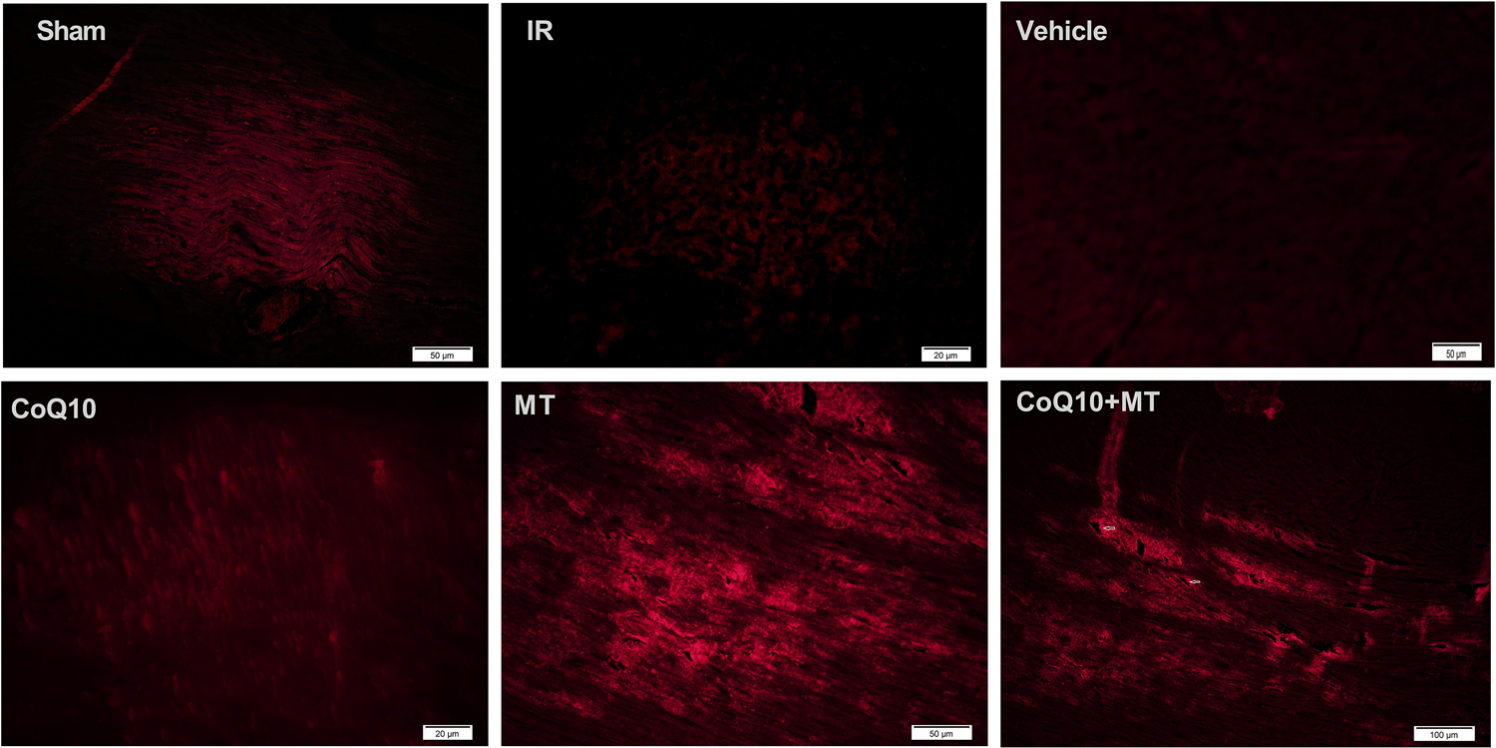
Cardiac tissue fluorescence microscopy images from different groups of aged male rats. All paraffin‐embedded sections indicated a little autofluorescence, which is obviously uniform in all parts of tissue. MT and MT + CoQ_10_ groups received MitoTracker Red‐labelled mitochondria. Shining red colour indicates transplanted mitochondria. The red fluorescence originated from vessels and entered into cells. Red fluorescence is more intense around vessels (white arrow). Magnification, ×200. CoQ_10_, coenzyme Q_10_; IR, ischaemia–reperfusion; MT, mitochondrial transplantation.

As shown in Figure 1, MitoTracker Red‐labelled mitochondria successfully entered cardiac tissue after injection, as evidenced by the red fluorescence emitted from the labelled mitochondria under a fluorescence microscope. All paraffin‐embedded sections exhibited a degree of autofluorescence, which was uniformly distributed throughout the tissue. However, upon injection of labelled mitochondria into the bloodstream, the red fluorescence emitted by MitoTracker was observed to originate from vessels and subsequently enter the cells. The red fluorescence intensity was notably more pronounced around vessels, as indicated by white arrows.

### 3.2 Haemodynamic parameters

**FIGURE 2:**
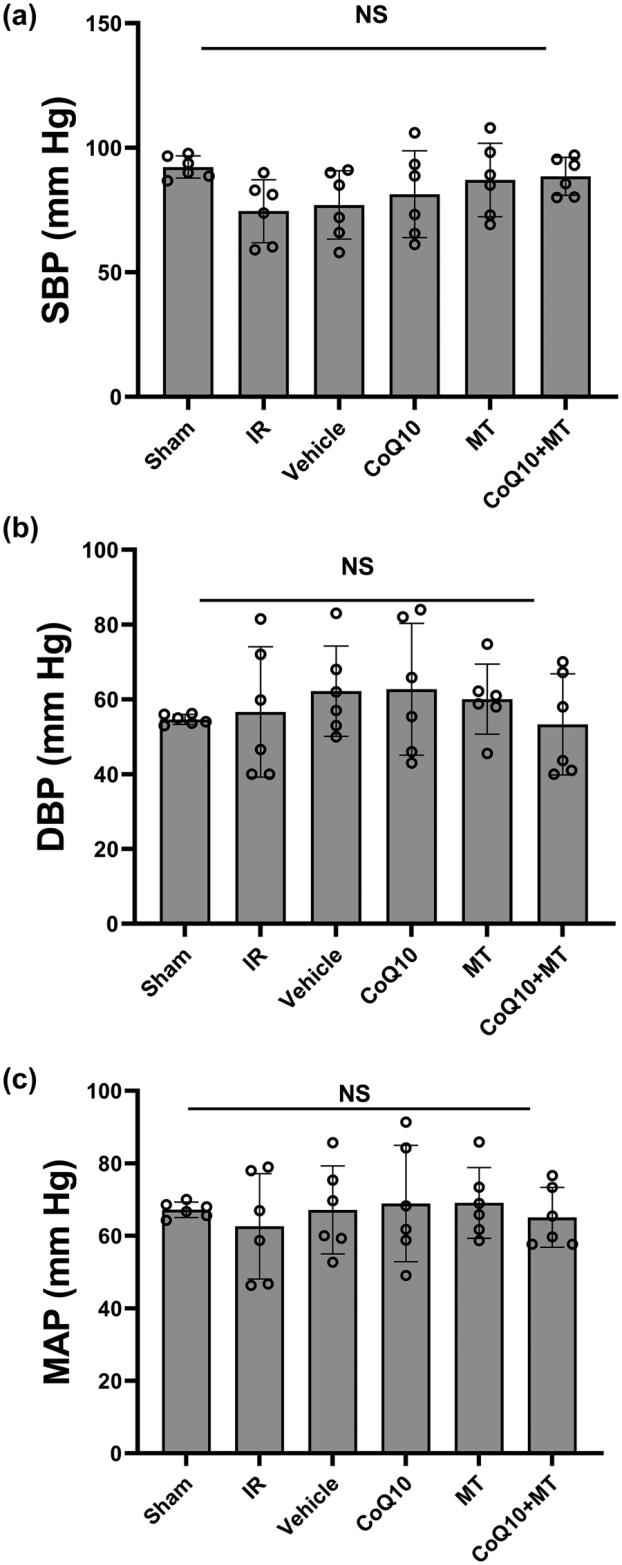
(a) Systolic blood pressure (SBP), (b) diastolic blood pressure (DBP), and (c) mean arterial pressure (MAP). *n* = 6 per group in aged male rats. Data are reported as means ± SD. CoQ_10_, coenzyme Q_10_; IR, ischaemia–reperfusion; MT, mitochondrial transplantation.

**FIGURE 3:**
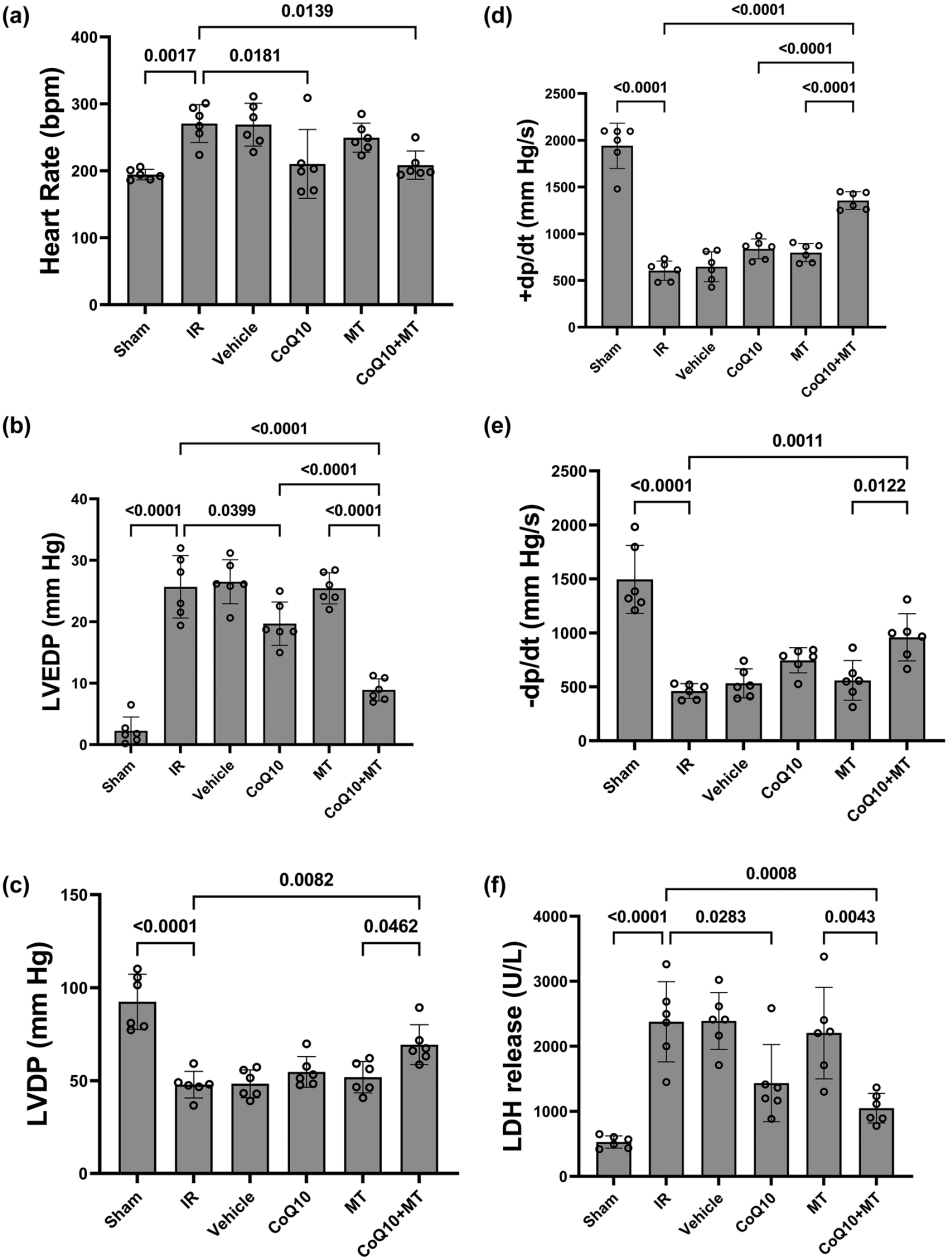
(a) Heart rate (HR), (b) left ventricular end‐diastolic pressure (LVEDP), (c) left ventricular developed pressure (LVDP), (d) maximum increase in left ventricular pressure alterations (+d*P*/d*t*), (e) maximum decrease in left ventricular pressure alterations (−d*P*/d*t*), and (f) level of lactate dehydrogenase (LDH) in aged male rats. *n* = 6 per group. Data are reported as means ± SD. CoQ_10_, coenzyme Q_10_; IR, ischaemia–reperfusion; MT, mitochondrial transplantation.

In the present study, arterial pressures including systolic and diastolic pressures and mean arterial pressure (MAP) were investigated. As shown in Figure 2, no significant differences were observed in the arterial pressures, SBP (*P* = 0.135), DBP (*P* = 0.740) and MAP (*P* = 0.921), between experimental groups. Also, the haemodynamic status of the heart including heart rate, LVEDP and LVDP were assessed (Figure 3). As shown in Figure 3a, a significant increase in the heart rate (HR) was observed in the IR group compared to the sham group (*P* = 0.0017). Treatment with CoQ_10_ as well as combination therapy significantly reduced HR compared to the IR group (*P* = 0.0181 and *P* = 0.0139, respectively). However, MT alone did not yield a significant reduction in HR in comparison to the IR group (*P* = 0.8250). Figure 3b shows that LVEDP in the IR group was significantly increased compared to the Sham group (*P* < 0.0001). Administration of CoQ_10_ as well as combination therapy significantly reduced LVEDP compared to the IR group (*P* = 0.0399 and *P* < 0.0001, respectively). However, the MT group did not show any significant difference from the IR group (*P* = 0.999). The effect of combination therapy on reducing LVEDP was significantly greater than CoQ_10_ alone (*P* < 0.0001). As shown in Figure 3c, LVDP was significantly decreased in the IR group compared to the Sham group (*P* < 0.0001), and only the combined treatment was able to significantly compensate for this decrease (*P* = 0.0082).

In order to check the contractility of the heart, the ±d*P*/d*t* index was measured in aged male rats across experimental groups (Figure 3d,e). The IR group exhibited a significant decrease in ±d*P*/d*t* compared to the Sham group (*P* < 0.0001). The combination therapy resulted in a significant increase in +d*P*/d*t* compared to the IR (*P* < 0.0001) and single treatment (*P* < 0.0001) groups. Additionally, a significant increase in −d*P*/d*t* was observed in the combination therapy group compared to the IR (*P* = 0.0011) and MT (*P* = 0.0122) groups (Figure 3d,e).

### 3.3 LDH release

The level of LDH release in the IR group was significantly higher than that in the Sham group (*P* < 0.0001). CoQ_10_ pretreatment significantly decreased this elevation compared to the IR group (*P* = 0.0283). However, the administration of mitochondria did not lead to any significant differences when compared to the IR group (*P* = 0.9897). Furthermore, the combination therapy significantly reduced LDH release in comparison to the IR (*P* = 0.0008) and MT (*P* = 0.0043) groups (Figure 3f).

### 3.4 Infarct size

**FIGURE 4:**
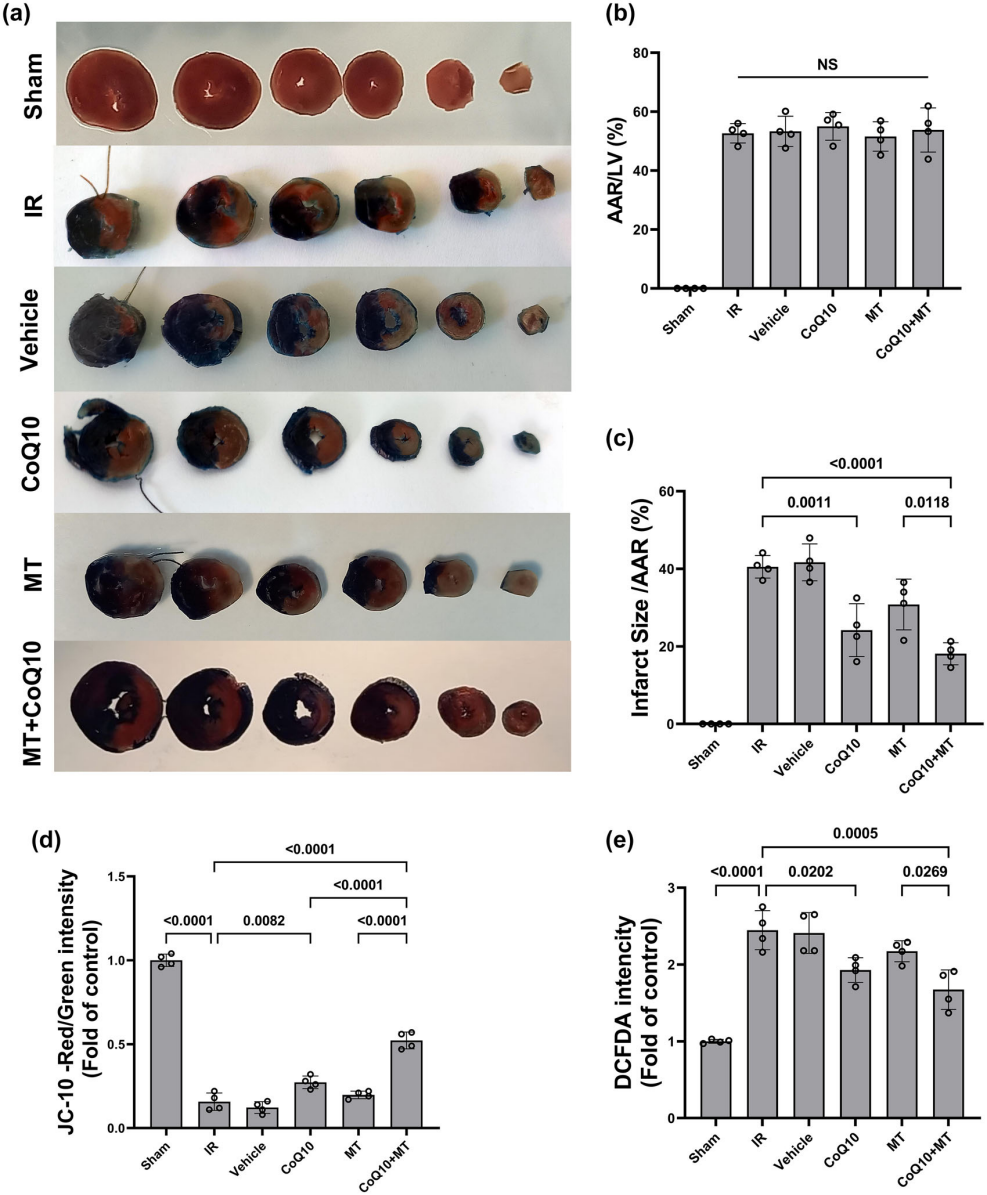
(a) Evans blue and TTC double staining of aged male rat hearts in each group. The infarcted myocardium is pale, the area at risk (AAR) including the viable myocardium is stained red, and the non‐ischaemic area is stained blue. (b) AAR expressed as a percentage of the total area. (c) Infarct size reported as a percentage of AAR. (d) Mitochondrial membrane potential (red/green JC‐10 ratio). (e) Mitochondrial ROS production (DCFDA intensity). *n* = 4 per group. Data are reported as means ± SD. CoQ_10_, coenzyme Q_10_; IR, ischaemia–reperfusion; MT, mitochondrial transplantation.

The AAR was the same between all experimental groups (Figure 4b). CoQ_10_ and combination treatment groups showed a significant decrease in the infarct size compared to the IR group (*P* = 0.0011 and *P* < 0.0001, respectively). However, single MT did not result in a significant decrease in infarct size compared to the IR group (*P* = 0.0751), and there was a significant difference between the combination therapy and MT groups (*P* = 0.0118) (Figure 4c).

### 3.5 Membrane potential

In this study, the ratio of red/green fluorescence intensity was measured as an indicator of mitochondrial membrane potential. The results showed that this ratio was significantly decreased in the IR group compared to the Sham group (*P* < 0.0001), which indicates mitochondrial membrane potential disruption (more depolarization). However, the ratio of red/green fluorescence intensity in the groups receiving CoQ_10_ as well as combination therapy was increased compared to the IR group (*P* = 0.0082 and *P* < 0.0001, respectively). However, single mitochondrial administration did not result in a significant difference compared to the IR group (*P* = 0.6502). Also, this ratio was significantly higher in the combination therapy group compared to the single treatment groups (*P* < 0.001) (Figure 4d).

### 3.6 Mitochondrial ROS production

The DCFDA intensity was measured as an indicator of ROS production. According to the results, the DCFDA intensity showed a significant increase in the IR group compared to the Sham group (*P* < 0.0001), which indicates a higher production of ROS. The DCFDA intensity in groups receiving CoQ_10_ as well as combination therapy was lower compared to the IR group (*P* = 0.0202 and *P* = 0.0005, respectively). Nevertheless, there was no significant difference between the MT and IR groups (*P* = 0.3501). Also, DCFDA intensity was significantly lower in the combination therapy group compared to the MT group (*P* = 0.0269) (Figure 4e).

## 4 DISCUSSION

In the present work, we explored the effects of CoQ_10_, a natural component of the mitochondrial system, when administered alone and in combination with MT in an in vivo model of aged male rats exposed to myocardial IR injury. The administration of CoQ_10_ demonstrated a degree of reduction in myocardial IR injury, but, the single administration of MT did not yield notable impacts on the outcomes of IR. However, the combination of CoQ_10_ and MT efficiently led to a considerable reduction in myocardial infarct size and LDH release, and resulted in positive changes in heart haemodynamics, including a decrease in LV diastolic pressure and an increase in developed pressure and cardiac contractility. The cardioprotective effect of this combination therapy was associated with increased mitochondrial membrane potential and decreased mitochondrial ROS production in the aged rats.

Many individuals who experience myocardial IR injury are elderly. Ageing brings about a range of unfavourable biological changes in cardiomyocytes, such as heightened production of ROS and oxidative stress, impaired autophagy, mitochondrial dysfunction, inflammation, metabolic dysregulation and cell death (Hosseini et al., 2019; Yan et al., 2021). Furthermore, the accumulation of damaged mitochondria in cardiomyocytes is a key feature of cardiac ageing (Hosseini et al., 2020). All of these detrimental changes can render the heart more susceptible to IR injuries and consequently diminish the effectiveness of single therapeutic modalities in the context of ageing. Therefore, our study aimed to explore whether a multi‐intervention approach could exert significant protective effects in aged hearts.

Pre‐administration of CoQ_10_ had some positive effects on aged IR hearts via improving mitochondrial function. Consistent with our results, previous studies conducted on healthy young animals without risk factors demonstrated significant cardioprotective effects of CoQ_10_ treatment against IR injury through various mechanisms. For instance, it has been found that CoQ_10_ reduced infarct size and enhanced cardiac function in animals exposed to acute myocardial IR injury by balancing myocardial oxidants and antioxidants, promoting autophagy and decreasing myocardial cell death (Khan et al., 2017; Liang et al., 2017). Eleawa et al. (2015) also documented that CoQ_10_ protects against acute experimental myocardial infarction in rats by increasing antioxidant enzymes, preventing DNA damage and inflammatory reactions. Moreover, CoQ_10_ treatment in optic nerve astrocytes showed significant potential for increasing mitochondrial mass and improving the bioenergetic status (Noh et al., 2013).

Although our study revealed some levels of protective effects of CoQ_10_ in aged rats, these effects were not as robust and reliable when compared to the group that received combination therapy. In essence, the protective effects of CoQ_10_ were not consistently observed across all cardiac parameters, suggesting that it may not provide complete protection to the aged heart against IR injury. As individuals age, there is a natural decline in the levels of endogenous CoQ_10_ and other essential substrates required for normal mitochondrial function (Aaseth et al., 2021). Consequently, supplementing with CoQ_10_ becomes necessary in old age. Importantly, selenium levels, which play a crucial role in the effectiveness of CoQ_10_, also decrease with ageing, potentially diminishing the efficacy of CoQ_10_ in IR conditions (Alehagen & Aaseth, 2015; Dunning et al., 2023). Furthermore, when CoQ_10_ is administered to older rats, it interacts with those mitochondria that are somewhat compromised and dysfunctional due to the ageing process. Consequently, to enhance the effectiveness of CoQ_10_ in the context of ageing, a combination therapy approach may be needed. Accordingly, it is reasonable to hypothesize that if healthy and functional mitochondria are transplanted into elderly rat hearts experiencing IR injury, CoQ_10_ could synergize with these healthier mitochondria, leading to a more robust protective effect.

MT, which involves transferring healthy and functional mitochondria to the cells with malfunctioning mitochondria, represents an innovative approach for addressing mitochondrial dysfunction. This approach aims to overcome the limitations of conventional therapeutic agents (Park et al., 2021). There is evidence to suggest that MT can confer significant protection in hearts with IR injury in young animal models (Cowan et al., 2016; Guariento et al., 2020; Kaza et al., 2017; Moskowitzova et al., 2019). A comprehensive study conducted by Masuzawa et al. showed that transplantation of autologous mitochondria into the IR hearts of New Zealand White rabbits led to a reduction of infarct size and myocardial damage markers. Furthermore, this therapy increased oxygen consumption, ATP production, cellular respiration and the release of cardioprotective cytokines, which play essential roles in angiogenesis, immune modulation and apoptosis prevention (Masuzawa et al., 2013). McCully et al. also reported that transplanting exogenous mitochondria into Langendorff‐perfused rabbit hearts at the onset of reperfusion had cardioprotective effects by improving cardiac haemodynamic status and reducing injury markers, including TUNEL‐positive cells and caspase‐3 activity (McCully et al., 2009). Despite the favourable cardioprotective results seen in prior studies involving MT for IR injury, our current investigation did not yield significant cardioprotective effects from MT as a standalone treatment. This disparity could be attributed to the fact that earlier studies primarily used young and healthy animal models without risk factors or comorbidities, while our study involved aged rats. Given the numerous changes in intracellular components during the ageing process, it is reasonable to assume that the transplanted mitochondria could not function optimally in this less favourable environment with altered homeostasis (Hartl, 2016). In other words, the natural process of ageing leads to a decrease in the levels of certain substances crucial for proper mitochondrial function, such as CoQ_10_, NAD^+^, and selenium (Aaseth et al., 2021; Alehagen & Aaseth, 2015; Hosseini et al., 2019). As a result, despite the transplantation of healthy and viable mitochondria, significant cardioprotective benefits were not evident in aged hearts.

Importantly, when CoQ_10_ was administered to aged rats prior to MT, the cardioprotection was stronger and more dependable. This combined treatment proved more effective in reducing myocardial infarct size, lowering LDH release and enhancing the heart's haemodynamic function. The cardioprotective advantages of this combined treatment were associated with improved mitochondrial activity, including reduced membrane depolarization and ROS generation. It appears that the cardioprotective effects of single treatments were less pronounced in the context of ageing. Therefore, it can be deduced that MT can also be protective, but its effects became apparent when the cellular environment was preconditioned with CoQ_10_. In this scenario, CoQ_10_, acting as a mitochondrial stimulant and enhancer, replenished intracellular reserves, creating a boosting setting for transplanted mitochondria to exert their protective effects.

MT in research on myocardial IR injury involves different injection sites. In various studies, injections have been administered either locally at specific points on the myocardium or through intravascular routes. Notably, our approach entails injecting into the left ventricular chamber, resembling a systemic bloodstream delivery. It is worth noting that with local injection, mitochondria tend to concentrate primarily at the injection site, and their spread to nearby areas is limited (Norat et al., 2023). In contrast, injection into the bloodstream increases the potential for mitochondrial migration, thereby promoting a more homogeneous distribution across all affected regions (Norat et al., 2023). Furthermore, the appeal of bloodstream delivery lies in its less invasive nature, which holds particular relevance when achieving myocardial reperfusion without thoracotomy, using methods like thrombolytic therapy or percutaneous coronary intervention. This presents a promising option for broader clinical applicability, wherein accessing the circulation offers a feasible and effective means of delivering therapeutic mitochondria.

### 4.1 Limitations and suggestions

In our study, the post‐MT observation period was relatively short, which limited our ability to assess the potential immune response to the transplanted mitochondria. Nonetheless, it appears unlikely that immune responses would occur within this brief timeframe. While previous studies have suggested that MT typically does not trigger an immune response, further investigations with longer observation periods are needed to confirm this finding.

Instead of intravenous injection, we opted for direct mitochondrial injection into the ventricle chamber immediately upon reperfusion when the heart was exposed to minimize invasiveness. This approach may limit the study's generalizability to a broader context.

We focused on the indices of mitochondrial function contributing to the cardioprotective effects of combination therapy in our study, but a more in‐depth exploration of mitochondrial respiration, fission/fusion and biogenesis as well as other survival mediators and mechanisms could strengthen our findings.

Our study exclusively focused on male aged rats. Considering age and sex as the biological variables in basic and clinical studies, including young and female rats and comparing their responses to those of the elderly could provide valuable insights into the effectiveness of the intervention in different sex and age groups.

We did not investigate whether CoQ_10_ pretreatment enhances mitochondrial internalization in this study. This aspect should be considered for future research investigations.

### 4.2 Conclusion

The findings from our present study demonstrated that the cardioprotective effect of combination therapy involving MT and CoQ_10_ surpassed that of their individual treatments and improved haemodynamic indices, decreased infarct size and LDH release, and improved mitochondrial function. As a result, it can be considered as a promising strategy for cardioprotection against myocardial IR injury in aged individuals.

## AUTHOR CONTRIBUTIONS

Reza Badalzadeh did the study design and supervised the whole project. Soleyman Bafadam performed the experimental tests, gathered and analysed the data, and wrote the manuscript. Zohreh Zavvari Oscuyi, Behnaz Mokhtari and Samira Nemati contributed in experimental tests. Reza Badalzadeh, Behnaz Mokhtari and Manoucheher Seyedi Vafaee revised and finalized the manuscript. All authors have read and approved the final version of this manuscript and agree to be accountable for all aspects of the work in ensuring that questions related to the accuracy or integrity of any part of the work are appropriately investigated and resolved. All persons designated as authors qualify for authorship, and all those who qualify for authorship are listed.

## CONFLICT OF INTEREST

The authors declare no conflicts of interest.

## Data Availability

The data are available from the corresponding author on reasonable request.
